# Sea Anemones Responding to Sex Hormones, Oxybenzone, and Benzyl Butyl Phthalate: Transcriptional Profiling and *in Silico* Modelling Provide Clues to Decipher Endocrine Disruption in Cnidarians

**DOI:** 10.3389/fgene.2021.793306

**Published:** 2022-01-11

**Authors:** Michael B. Morgan, James Ross, Joseph Ellwanger, Rebecca Martin Phrommala, Hannah Youngblood, Dominic Qualley, Jacob Williams

**Affiliations:** ^1^ Department of Biology, Berry College, Mount Berry, GA, United States; ^2^ Department of Chemistry and Biochemistry, Berry College, Mount Berry, GA, United States; ^3^ Department of Microbiology and Immunology, Emory Vaccine Center, Emory University School of Medicine, Atlanta, GA, United States; ^4^ Department of Cellular Biology and Anatomy, Augusta University, Augusta, GA, United States

**Keywords:** endocrine disruption chemicals, cnidaria, in silico modelling and docking, biomarkers, sex hormones, xenobiotics, transcriptional profiling, hedgehog signaling

## Abstract

Endocrine disruption is suspected in cnidarians, but questions remain how occurs. Steroid sex hormones are detected in corals and sea anemones even though these animals do not have estrogen receptors and their repertoire of steroidogenic enzymes appears to be incomplete. Pathways associated with sex hormone biosynthesis and sterol signaling are an understudied area in cnidarian biology. The objective of this study was to identify a suite of genes that can be linked to exposure of endocrine disruptors. *Exaiptasia diaphana* were exposed to nominal 20ppb concentrations of estradiol (E2), testosterone (T), cholesterol, oxybenzone (BP-3), or benzyl butyl phthalate (BBP) for 4 h. Eleven genes of interest (GOIs) were chosen from a previously generated EST library. The GOIs are *17β-hydroxysteroid dehydrogenases type 14* (*17β HSD14*) and *type 12* (*17β HSD12*), *Niemann-Pick C type 2* (*NPC2*), *Equistatin* (*EI*), *Complement component C3* (*C3*), *Cathepsin L* (*CTSL*), *Patched domain-containing protein 3* (*PTCH3*), *Smoothened* (*SMO*), *Desert Hedgehog* (*DHH*), *Zinc finger protein GLI2* (*GLI2*), and *Vitellogenin* (*VTG*). These GOIs were selected because of functional associations with steroid hormone biosynthesis; cholesterol binding/transport; immunity; phagocytosis; or Hedgehog signaling. Quantitative Real-Time PCR quantified expression of GOIs. *In silico* modelling utilized protein structures from Protein Data Bank as well as creating protein structures with SWISS-MODEL. Results show transcription of steroidogenic enzymes, and cholesterol binding/transport proteins have similar transcription profiles for E2, T, and cholesterol treatments, but different profiles when BP-3 or BBP is present. *C3* expression can differentiate between exposures to BP-3 versus BBP as well as exposure to cholesterol versus sex hormones. *In silico* modelling revealed all ligands (E2, T, cholesterol, BBP, and BP-3) have favorable binding affinities with 17β HSD14, 17β HSD12, NPC2, SMO, and PTCH proteins. *VTG* expression was down-regulated in the sterol treatments but up-regulated in BP-3 and BBP treatments. In summary, these eleven GOIs collectively generate unique transcriptional profiles capable of discriminating between the five chemical exposures used in this investigation. This suite of GOIs are candidate biomarkers for detecting transcriptional changes in steroidogenesis, gametogenesis, sterol transport, and Hedgehog signaling. Detection of disruptions in these pathways offers new insight into endocrine disruption in cnidarians.

## Introduction

In the past quarter century, public awareness of the dramatic declines in coral reefs has become increasingly evident. Extensive research has characterized how coral reefs are shifting in structure and biodiversity due to climate change, over-fishing, coastal development, and pollution (Gardner et al., 2003; Hughes et al., 2003; Pandolfi et al., 2003; Jones et al., 2004; Bruno and Selig, 2007). Understanding the impact of land-based pollution onto reefs is further complicated by pulses of anthropogenic activity, variations in tides, variations in seasonal precipitation, and sediments which can act as carriers for a variety of organic compounds (Dachs et al., 1999; Gavio et al., 2010; Singh et al., 2010; Burns and Brinkman, 2011; Edge et al., 2013). Both land-based pollution and sewage discharge produce terrestrial runoff which culminates in diminished water quality in coastal marine environments (McKenna et al., 2001; Fabricius, 2005; Gavio et al., 2010; Singh et al., 2010; Vidal-Dorsch et al., 2012; Bahr et al., 2015; French et al., 2015; Wear and Thurber, 2015; Yoshioka et al., 2016; Al-Jandal et al., 2018). The composition of sewage is a complex mixture of freshwater, heavy metals, inorganic nutrients, microplastics, pathogens, pesticides, pharmaceuticals, plasticizers, sediments, suspended solids, and toxins (Jones et al., 2003; Kerswell and Jones, 2003; Flood et al., 2005; Jones, 2010; Singh et al., 2010; Sutherland et al., 2010; Tijani et al., 2013; French et al., 2015; Mahon et al., 2017). Many components of sewage are recognized as endocrine disrupting chemicals (EDCs) whose impact on marine invertebrates needs to be characterized (Tan et al., 2007; Singh et al., 2010; Wear and Thurber, 2015).

One form of pollution in the marine environment that is ubiquitous even in sewage is plastic (Law, 2017; Mahon et al., 2017; Lebreton et al., 2018). Plastics are known to accumulate and persist longer in sediments than on land (Worm et al., 2017). With the global explosion of plastic pollution, there is evidence cnidarians are capable of ingesting microplastics which are known to release phthalates (Hall et al., 2015; Axworthy and Padilla-Gamiño, 2019; Rotjan et al., 2019; Deng et al., 2020). As a leachable compound from plastics, phthalates are one of the most frequently detected persistent organic pollutants in the environment (Gao and Wen, 2016). In addition to plastics, oxybenzone is becoming an increasingly significant environmental concern for coral reefs because it has been detected in coastal surface waters and sediments, and has been shown to influence larval development (Downs et al., 2016; Mitchelmore et al., 2019). Steroid sex hormones, phthalates, and oxybenzone are all recognized as EDCs and all have been detected in sewage effluent and coastal marine environments where anthropogenic activity is prevalent (Atkinson et al., 2003; Thibaut and Porte, 2004; Singh et al., 2010; Bargar et al., 2013; Tsui et al., 2014; French et al., 2015; Thorn et al., 2015; Al-Jandal et al., 2018; LaPlante et al., 2018; Matouskova et al., 2020). Sterols represent the second highest proportion of lipids (14–17%) found in cnidarians (Revel et al., 2016). Because steroid hormones, phthalates, and oxybenzone all have lipophilic characteristics, the lipid-rich tissues of cnidarians are likely targets for EDCs. It has been proposed that lipophilic compounds will easily diffuse into cnidarian tissues and bioaccumulate (Peters et al., 1997; Imbs, 2013; Ko et al., 2014). Using a variety of mass-spectrometry assays, steroid hormones (and/or their conjugates), phthalates, and oxybenzone have been detected in coral tissues (from <10 ng up to 650 ng g^−1^ dry weight) and/or the environment (from <10 ng L^−1^ to >100 μg L^−1^), (Solbakken et al., 1985; Tarrant et al., 2003; Twan et al., 2003; Twan et al., 2006; Downs et al., 2016; Mitchelmore et al., 2019). Cnidarians have demonstrated the ability to uptake estrogens in the water column at concentrations as low as 300 pg/L (Tarrant et al., 2001).

Steroid sex hormones are known to be important in cnidarian development and reproduction (Atkinson and Atkinson, 1992; Gassman and Kennedy, 1992; Slattery et al., 1999; Tarrant et al., 1999; Twan et al., 2003; Twan et al., 2006; Armoza-Zvuloni et al., 2014). Exogenous estrogen is known to impact asexual reproduction (Thorn et al., 2015). A conundrum exists because anthozoans do not have nuclear estrogen receptors even though the hormone is detectable in tissues and functions in their reproductive biology (Reitzel et al., 2008; Reitzel and Tarrant, 2009). Nuclear receptors capable of binding estrogen have been identified in other cnidarians (medusozoans) (Khalturin et al., 2018), and although not yet well-characterized, a membrane-bound G-protein coupled estrogen receptor 1-like transcript (GPER1) genomic locus (LOC114576065) has been identified for *E. diaphana* (NCBI Nucleotide). GPER1 has recently been identified in humans in addition to the two traditional nuclear estrogen receptors ERα and ERβ (Fuentes and Silveyra, 2019). Perhaps GPER1 is also involved in physiologic estrogen-signaling in anthozoans and may be a target of EDCs, leading to downstream transcriptional changes.

Another conundrum exists because cnidarians are known to synthesize a diverse set of sterols (Tarrant et al., 2009; Revel et al., 2016), but their repertoire of steroidogenic enzymes appears to be incomplete. There is no genomic evidence that cnidarians produce aromatase, and yet aromatase activity has been detected (Tarrant et al., 2003; Twan et al., 2003; Twan et al., 2006). It is also possible that steroid sex hormones may play other significant roles (e.g., possibly chemical communication) which have yet to be fully elucidated (Tarrant et al., 2003; Blomquist et al., 2006; Lafont and Mathieu, 2007; Shikina et al., 2016). Since cnidarians use steroid hormones as signaling molecules but they do not have an endocrine system, Tarrant (2007) asked a fundamental question: Can endocrine disruption occur in cnidarians? Previous investigations have identified steroid hormone-like signaling molecules but there are still significant gaps in understanding which metabolic pathways are impacted.

One approach of ecotoxicological investigations is to identify changes in gene transcription in aquatic organisms exposed to an exogenous substance (Snell et al., 2003; Snape et al., 2004). Calls have been made to develop a comprehensive assessment of steroid hormones, phthalates, and personal care products impacting marine environments (Singh et al., 2010; Tijani et al., 2013; Archer et al., 2017; DiNardo and Downs 2018). More detailed information regarding the impact of EDCs on specific pathways involved in both synthesis and metabolism of sex hormones would be highly desirable. Developing biomarkers for anthropogenic stressors can be useful tools for monitoring health on coral reefs (Kenkel et al., 2014). Validated mechanistic biomarkers for aquatic invertebrates which can identify responses to a particular class of chemical stressor a have been theorized (Snell et al., 2003; Hutchinson, 2007), but difficult to produce. Presently, biomarkers for cnidarians exposed to EDCs have not been developed and validated. This study seeks to identify a suite of genes that characterize exogenous EDC exposures. Such a suite of genes could help elucidate the cellular pathways leading to cnidarian endocrine disruption.

## Materials and Methods

### Toxicant Exposure

Sea anemones (*Exaiptasia diaphana* previously known as *Aiptasia pallida*) were purchased from a supplier (Wards Natural Science, Rochester, NY, United States) and acclimated to laboratory conditions (45 L recirculating artificial seawater at 22°C and 36 ppt salinity) for 1 week. Anemones (100 individuals) were randomly subdivided into a control group or 20 ppb treatments of 17β-estradiol (E2), testosterone [T], cholesterol, benzyl butyl phthalate (BBP), or oxybenzone (BP-3). Toxicant purity was pharmaceutical secondary standard grade for E2, T, cholesterol, and BP-3 while BBP was analytical standard grade (Sigma-Aldrich, St. Louis, MO, United States). Acetone (150 μg/L) was used to solubilized toxicants were prior to dilution in seawater. Acetone has previously been used as a wetting agent (Morgan et al., 2001; Morgan et al., 2012). The 20 ppb concentration was chosen to induce responses and does not necessarily reflect environmental conditions even though BP-3 has been detected at concentrations >20 ppb (Downs et al., 2016) and cholesterol at 11ppb (French et al., 2015). All treatments were nominal concentrations for 4 h in 1L artificial seawater under ambient laboratory lighting during the early spring season. The 4-h exposure was chosen to detect responses at an early stage of exposure.

### Selecting Candidate Genes of Interest

Complementary DNA sequences previously generated by Representational Difference Analysis (RDA) (Edman et al., 1997; Hubank and Schatz, 1999; Pastorian et al., 2000) were screened for candidate ESTs to be used in this investigation. Candidate ESTs were selected based on sequence homology (BLASTX analysis) to genes of interest (GOI) with known functions involving sex hormone synthesis, sterol binding, lipid transport, and/or indirect sterol interactions. Subsets of these ESTs have previously been used to characterize cnidarian stress responses (Morgan et al., 2012; Morgan et al., 2015).

### Reverse Transcription Reactions

Anemones from each treatment were pooled and total RNA was isolated using Trizol (Invitrogen, United States). Two milliliter phase-lock gels (5′Prime, United States) aided in the recovery of the aqueous phase. Total RNA was DNase I digested and purified (New England BioLabs, United States). Messenger RNA (mRNA) was isolated (Oligotex, Qiagen, United States) from 100 μg of DNase I treated total RNA. First stand synthesis used SuperScript IV (Invitrogen, United States) along with random hexamers and oligo-dT primers to reverse transcribe 1 μg of poly-A enriched mRNA. The RT reaction conditions were 1 h at 37°C, followed by 1 min at each temperature between 42 and 50°C.

### Quantitative Real-Time PCR

A QuantStudio 7 Flex Real-Time PCR system (Applied Biosystems Waltham, MA, United States) used a SYBR Green-based assay to perform qPCR. A 1/100 dilution of first-strand synthesis reactions were used as templates for all qPCR reactions. Primers for each GOI were created using Primer3 (https://primer3.org) (Table 1). Components for each 20 µl qPCR reaction included: 10 µl Luna^®^ Universal qPCR Mix (New England BioLabs, United States), 2.5 µl Forward primer (10 µM), 2.5 µl Reverse primer (10 µM), 2.5 µl dH_2_O, 2.5 µl sample. Thermocycling conditions were 1 cycle at 95°C for 1 min; 40 cycles of 95°C for 15 s and then 60°C for 30 s; and concluding with 1 cycle of melt curve analysis. Four replicate reactions were used for analyzing the relative expression of each GOI. Ribosomal protein L11 (*RPL11*) was used as qPCR reference gene (Kenkel et al., 2011). Melt-curve analysis, primer efficiencies, and gel electrophoresis confirmed specificity of priming. Replicate Cq values were averaged to determine ∆Cq and ∆∆Cq for each treatment and GOI. All ∆Cq and ∆∆Cq values are based on the consistent expression of *RPL11* across all treatments. The ∆∆Cq method was used to determine the differences between targeted GOIs and a single reference gene (Bustin et al., 2009). Univariate ANOVA was performed on ∆∆Cq data to identify significant expression of individual GOIs across treatments. Similarities in variance between treatments were determined by Levene’s Test of Equality of Error Variances. If the variance between treatments was similar, then the Student-Neuman-Keuls (SNK) posthoc test was performed to determine which treatment(s) were significantly different from the rest. If variance between treatments was different, then Tamhane’s T2 posthoc test was applied since Univariate ANOVA is generally insensitive to heteroscedasticity.

**TABLE 1:** BLAST results for RDA probes. Searches performed at NCBI using BLASTX algorithm and the non-redundant database (nr) with default search parameters.

Gene ID	Putative Gene Homolog	BLASTX	Organism ID	Homolog Accession #
		E-value		
17βHSD14	17β-hydroxysteroid dehydrogenase type 14	5e-155	Exaiptasia diaphana	KXJ20962
17βHSD12	17β-hydroxysteroid dehydrogenase type 12	1e-61	Exaiptasia diaphana	KXJ12187
C3	Complement component C3	3e-57	Exaiptasia diaphana	KXJ11955
CTSL	Cathepsin L	6e-95	Exaiptasia diaphana	KXJ27439
NPC2	Niemann-Pick C type 2	1e-84	Exaiptasia diaphana	KXJ29862
EI	Equistatin	7e-57	Exaiptasia diaphana	KXJ18222
PTCH3	Patched domain-containing protein 3	3e-79	Exaiptasia diaphana	KXJ20037
DHH	Desert hedgehog protein A	3e-47	Exaiptasia diaphana	KXJ11164
SMO	Smoothened-like	1e-87	Exaiptasia diaphana	KXJ11374
GLI2	zinc finger protein GLi2	4e-146	Exaiptasia diaphana	XP_020898684
VTG	Vitellogenin 2	7e-38	Exaiptasia diaphana	KXJ14544

### In silico Molecular Modelling

Human protein homologs have previously been used as substitutes for cnidarian proteins in molecular modelling (Khalturin et al., 2018). Some cnidarian proteins have such significant homology to human proteins that they are even capable of stimulating human responses (Dunn et al., 2006; Wood-Charlson and Weis, 2009; Duffy and Frank, 2011; Dani et al., 2014; Brennan et al., 2017; Mansfield et al., 2017). The most similar vertebrate structures (according to BLASTX E-value) were used for selecting which Protein Databank (PDB) files should be used for initial docking experiments. Sequence alignments of cnidarian and relevant homologs determined which relevant protein sequences (i.e., PDB files) were to be used as representatives of corresponding cnidarian proteins. Protein coordinates (PDB files) for homologous constructs were downloaded from the PDB, and any existing ligands or small molecules (e.g., water or salt ions) were removed. Cnidarian protein structures were created with the SWISS-MODEL web server (https://swissmodel.expasy.org) using the homologs as templates. Ligands were created in Avogadro (Hanwell et al., 2012) and energy-minimized prior to docking. Docking was performed using AutoDock Vina (Trott and Olson, 2010); the search space was restricted to the known ligand binding site. After docking, the results were analyzed in UCSF Chimera (Pettersen et al., 2004) with the Find Clashes/Contacts function which was set to detect any atoms within 0.4 Å of the ligand with the best docking score.

## Results

### Genes of Interest

BLASTX analysis of ESTs identified eleven candidate GOIs with of functions associated with steroidogenesis, gametogenesis, cholesterol transport, immunity, phagocytosis, or the Hedgehog signaling pathway (Table 2). These GOIs were: 17β-hydroxysteroid dehydrogenase type 14 (*17βHSD14*), 17β-hydroxysteroid dehydrogenase type 12 (*17βHSD12*), Niemann-Pick C type 2 (*NPC2*), Equistatin (*EI*), Complement component C3 (*C3*), Cathepsin L (*CTSL*), Patched domain-containing protein 3 (*PTCH3*), Smoothened (*SMO*), Desert Hedgehog protein A (*DHH*), zinc finger protein Gli2 (*GLI2*), and Vitellogenin (*VTG*). Two GOIs (*17βHSD14*; *17βHSD12*) are associated with steroidogenesis. *17βHSD14* converts E2 into E1 and T into androstenedione (A4), while *17βHSD12* performs the reverse reactions, E1 into E2, and A4 into T (Tarrant et al., 2003; Luu-The et al., 2006; Shikina et al., 2016; Salah et al., 2019). Three GOIs (*CTSL, EI, NPC2*) are associated with lysosome/endosomes and/or symbiosome/phagosome (Dani et al., 2014; Mohamed et al., 2016; Dani et al., 2017; Hambleton et al., 2019). One GOI (C3) is associated with innate immunity (Miller et al., 2007; Kvennefors et al., 2010; Palmer and Traylor-Knowles, 2012; Ocampo et al., 2015). Four GOIs (*PTCH3, SMO, DHH, GLI2*) are representative of Hedgehog (HH) signaling which is highly conserved within vertebrate mammals (Sinkovics, 2015). One GOI (*VTG*) is the precursor of the egg yolk protein vitellin (Matozzo et al., 2008; Shikina et al., 2013; Lotan et al., 2014).

**TABLE 2:** Binding affinities (in kcal/mol) of ligands to proteins, estimated by docking simulations. Values represent the lowest energy binding mode for each docking experiment. Cnid: cnidarian; CS: crystal structure. Total number of amino acids and the total number of physical contacts represent binding pocket interactions. * indicates no direct homolog for PTCH3 so PTCH1 PDB file (6OEU) was used.

Gene ID	Ligand	Binding energy (kcal/mol)		Binding pocket	
				Total # of aa/total # contacts	
		Cnid	CS	Cnidarian (accession #)	Crystal structure (PDB file)
17βHSD14	Estradiol	−8.8	−8.4	16aa/238 (KXJ20962)	17aa/243 (5HS6)
	Testosterone	−8	−8.5		
	Cholesterol	−7.5	−6.4		
	BBP	−7.8	−7.2		
	BP-3	−7.4	−7.4		
17βHSD12	Cholesterol	−6.9	−7.4	17aa/217 (KXJ12187)	22aa/202 (2ET6)
	Estradiol	−6.4	−7.5		
	Testosterone	−6.4	−7.3		
	BBP	−5.2	−6.1		
	BP-3	−5.8	−5.7		
PTCH3*	Cholesterol	−9.4	−7.3	25aa/281KXJ20037	14aa/2136OEU
	Estradiol	−7.9	−7.8		
	Testosterone	−8.5	−8.1		
	BBP	−6.9	−6.2		
	BP-3	−6.6	−7.0		
NPC2	Cholesterol	−9.5	−12.1	18aa/177KXJ29862	19aa/2375KWY
	Estradiol	−8.5	−10.6		
	Testosterone	−8.7	−10.3		
	BBP	−6.9	−8.5		
	BP-3	−6.6	−8.0		
SMO	Cholesterol	−8.4	−9.9	12aa/190KXJ11374	20aa/2106XBM
	Estradiol	−7.6	−9.0		
	Testosterone	−8.2	−9.2		
	BBP	−6.6	−7.9		
	BP-3	−6.5	−7.5		

### Transcriptional Responses of Genes of Interests

**FIGURE 1:**
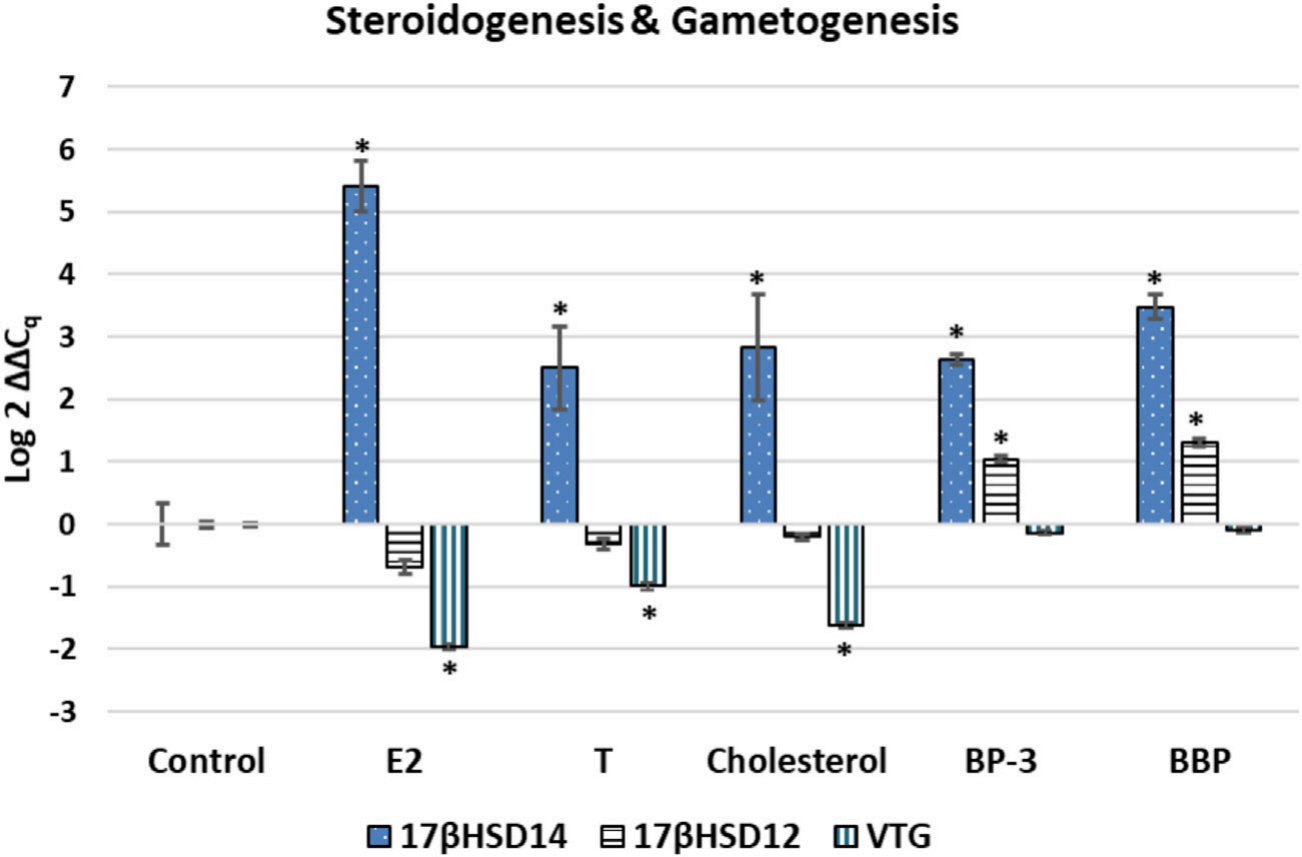
Expression profile for transcripts associated with sex hormone steroidogenesis or gametogenesis. The ∆∆Cq values represent transformed expression of a GOI relative to RPL11 expression. An * represents a treatment that was significantly different (*p* < 0.01) relative to the control condition. Error bars represent ± SE. E2: estradiol; T: testosterone; BP-3: oxybenzone; BBP: benzyl butyl phthalate; l7§HSD14: 17a-hydroxysteroid dehydrogenase type 14; 17bHSD12: 17b-hydroxysteroid dehydrogenase type 12: VTG: Vitellogenin 2

For the GOIs associated with steroidogenesis, *17βHSD14* expression was significantly up-regulated (*p* < 0.01) in each exogenous chemical treatment compared to the control, whereas *17βHSD12* expression was significantly up-regulated (*p* < 0.01) only in the BP-3 and BBP treatments (Figure 1). *VTG* exhibited significant down-regulated expression (*p* < 0.01) in E2, T, and cholesterol treatments (see Figure 1).

**FIGURE 2:**
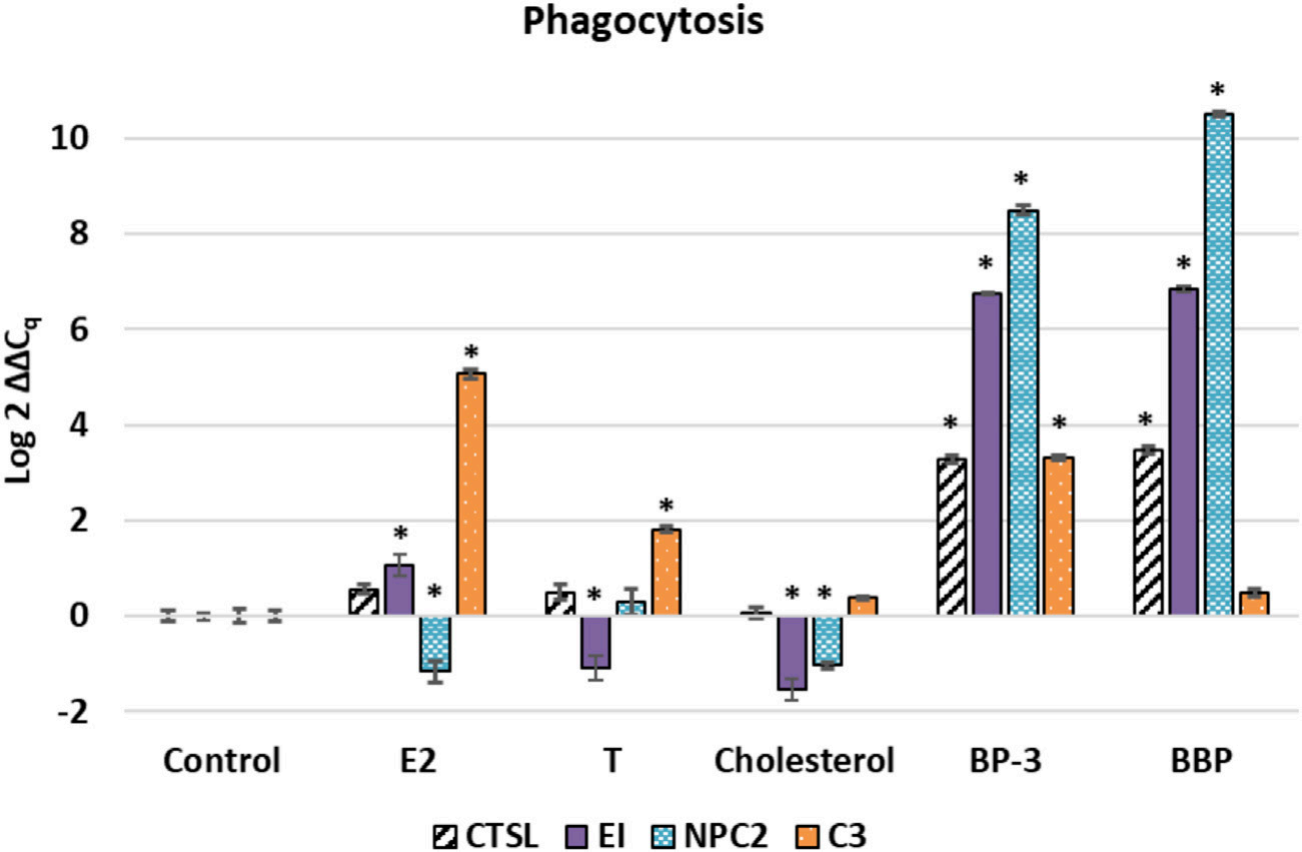
Expression profile of transcripts associated with phagocytosis and cellular structures such as symbiosome, lysosome, or endosome. The ∆∆Cq values represent transformed expression of a GOI relative to RPL11 expression. An * represents a treatment that was significantly different (*p* < 0.01) relative to the control condition. Error bars represent ± SE. E2: estradiol; T: testosterone; BP-3: oxybenzone; BBP: benzyl butyl phthalate. CSTL: cathepsin L; EI: equistatin; NPC2: Niemann-Pick C type 2; C3: complement component C3.

GOIs associated with phagocytosis had significant differences in expression in various treatments when compared to the control condition (Figure 2). Cathepsin (*CTSL*) expression was significantly up-regulated in the BP-3 and BBP treatments (*p* < 0.01). Equistatin (*EI*) was significantly up-regulated in E2, BP-3, and BBP treatments (*p* < 0.01) and significantly down-regulated in the T and cholesterol treatments (*p* < 0.01) (see Figure 2). *NPC2* was significantly down-regulated in cholesterol (*p* < 0.01) and significantly up-regulated (*p* < 0.01) in BP-3 and BBP treatments (see Figure 2). Lastly, *C3* was significantly up-regulated in E2, T, and BP-3 treatments (see Figure 2).

**FIGURE 3:**
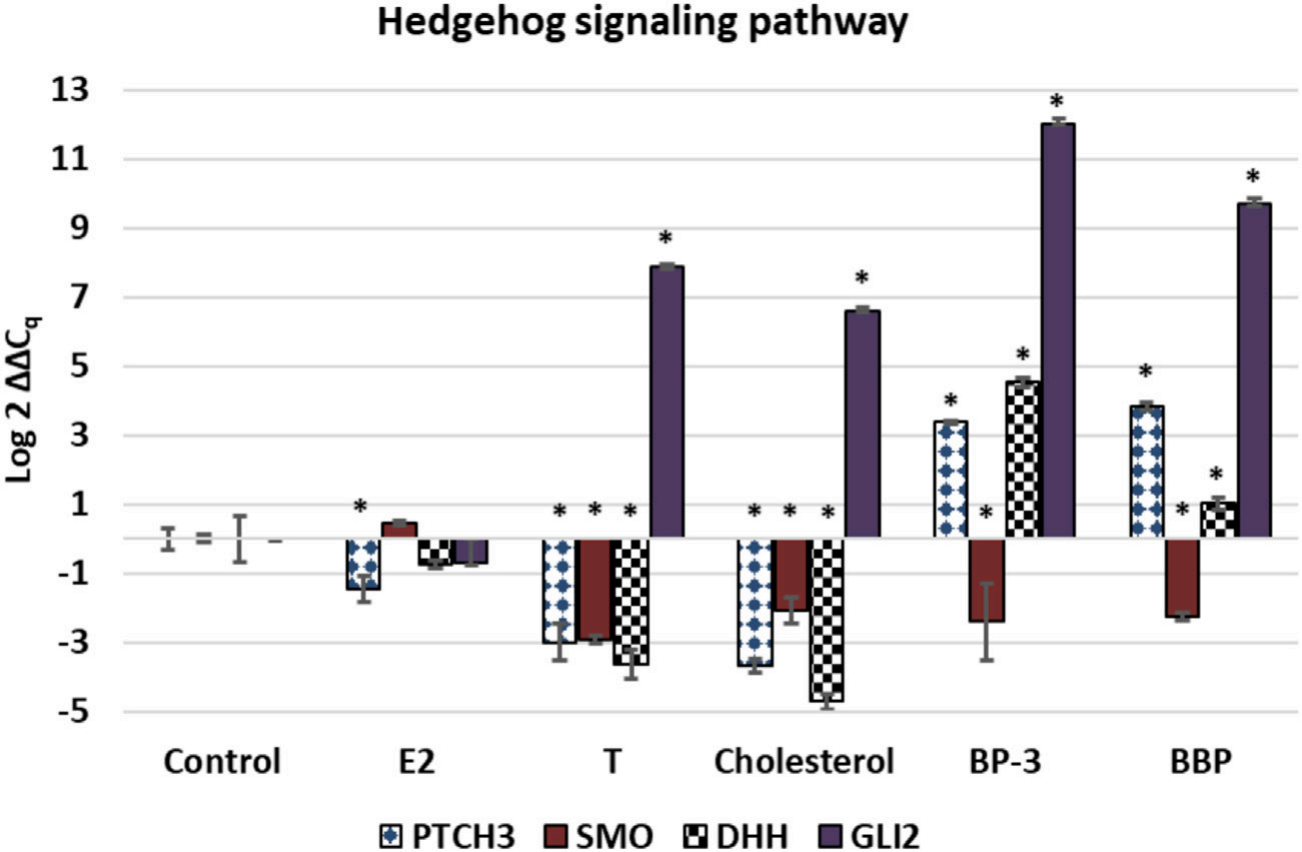
Expression profiles of transcripts associated with Hedgehog signaling pathway. The ∆∆Cq values represent transformed expression of GOI relative to RPL11 expression. An * represents a treatment that was significantly different (*p* < 0.01) relative to the control condition. Error bars represent ± SE. E2: estradiol; T: testosterone; BP-3: oxybenzone; BBP: benzyl butyl phthalate. PTCH3: patched domain-containing protein 3; SMO: smoothened-like; DHH: Desert hedgehog protein A; GLI2: zinc finger protein GLi2.

For the GOIs associated with HH signaling, *PTCH3* was significantly down-regulated (*p* < 0.01) in response to all three sterols, but significantly up-regulated (*p* < 0.01) in BP-3 and BBP treatments (Figure 3). *SMO* was significantly down-regulated (*p* < 0.01) in all treatments except E2 (see Figure 3). *DHH* was significantly down-regulated (*p* < 0.01) in T and cholesterol treatments, but significantly up-regulated (*p* < 0.01) in BP-3 and up-regulated in BBP treatments (*p* < 0.05) (see Figure 3). *GLI2* was significantly up-regulated (*p* < 0.01) in all treatments except E2 (see Figure 3).

**FIGURE 4:**
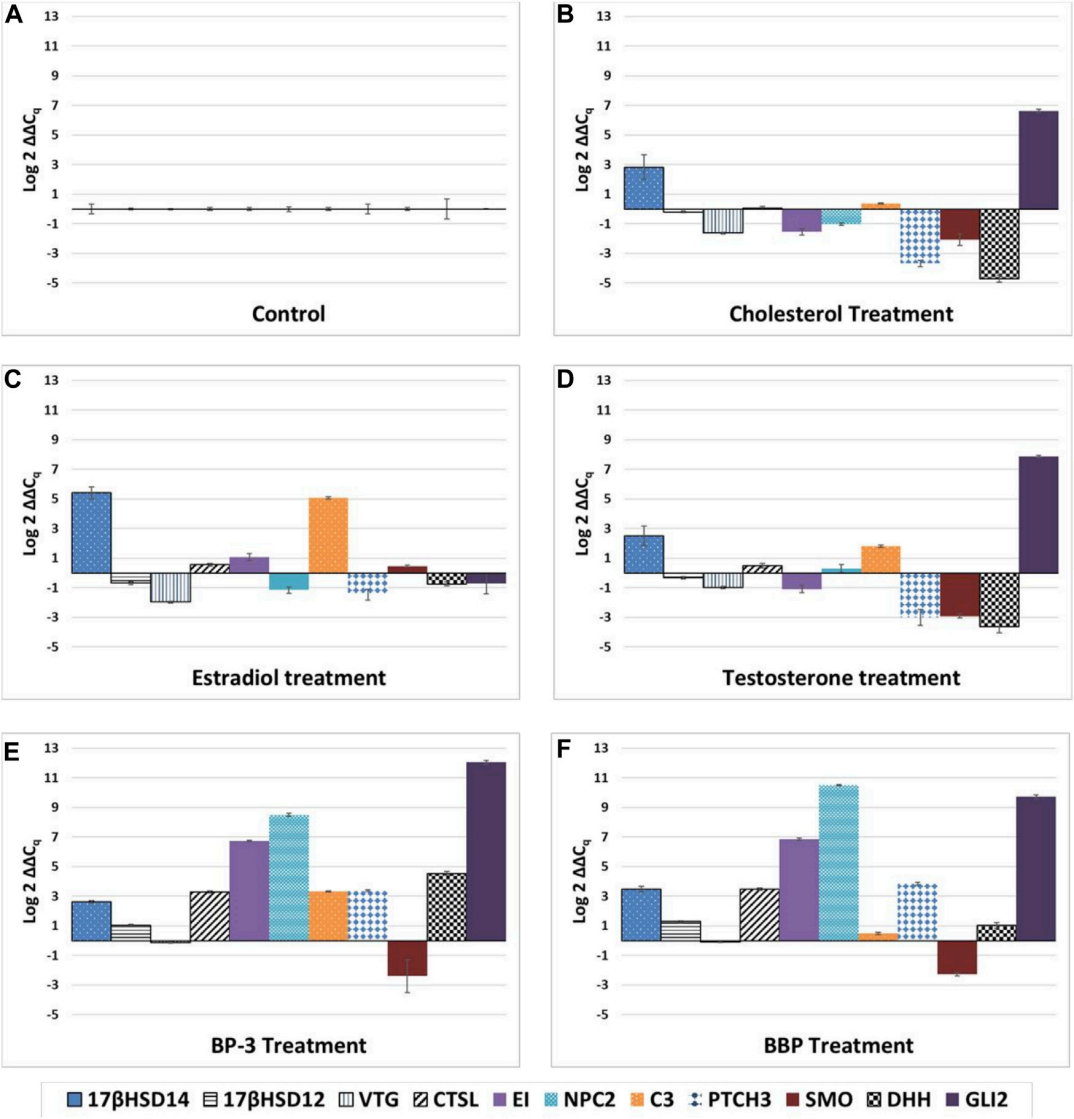
Unique expression profiles for each treatment. **(A)** control, **(B)** cholesterol, **(C)** estradiol, **(D)** testosterone, **(E)** oxybenzone, **(F)** benzyl butyl phthalate. The ∆∆Cq values represent transformed expression of GOI relative to RPL11 expression. Error bars represent ± SE.

Univariate ANOVA reveals all sterol treatments were significantly different from each other as well as the xenobiotics (*p* < 0.01). While the BP-3 and BBP treatments were significantly different from all the sterols (*p* < 0.01), they were not significantly different from each other (Figure 4).

### Docking Simulations

In order to better understand how sterols and xenobiotics are able to interact with targeted proteins, *in silico* modelling was used to predict the most energetically favorable ligand/protein binding interactions. The binding affinities of each ligand to the proteins of interest were estimated using docking simulations. Comparative genomic analyses indicate that cnidarian genomes show important similarities to vertebrates in gene content, genomic structure, and organization (Putnam et al., 2007). However, the cnidarian proteome is not as well characterized as the human proteome. Therefore, macromolecular structures needed for the *in silico* modelling were generated in one of two ways. The first model was the structure in the Protein Databank with the highest degree of homology to the GOI. These PDB files were: 6oeu (PTCHD3), 5kwy (NPC2), 6xbm (SMO), 5hs6 (17βHSD14), and 2et6 (17βHSD12). The best PDB structures corresponded to the human sequences in all cases except for 17βHSD12, where the protein with the closest homology was from *Candida tropicalis*. The second model was generated using SWISS-MODEL, with the *Exaiptasia* sequence as input and the closest PDB structure as a template. Each ligand was docked to both protein models. The docking algorithm used flexible fitting with an energy-minimized ligand to determine possible binding conformations and relative binding affinities. All ligands bind to each protein, although the strength of the interactions varied. The binding free energy of the best docking pose for each ligand/receptor combination is shown in Table 3.

**TABLE 3:** Genes of Interest and their corresponding primers used in qPCR reaction. Annealing temperature for all primers was 60°C.

		
Gene ID	Primers	Amplicon length
17βHSD14	F: TGC​ACC​CTT​TGT​TGT​GAC​AT	209bp
	R: GAT​GGC​ATC​CTC​CAG​AAA​GA	
17βHSD12	F: AGT​CCA​GAT​TTT​CTT​GCA​ACC​A	226bp
	R: TAG​ACT​TCA​GTG​GTG​GGC​AG	
VTG	F: GCT​GTA​GTG​GTT​TTG​GTC​GG	198bp
	R: TGG​TGC​TTC​TTG​GCT​TGT​TC	
CTSL	F: CAT​TGC​CAT​TGC​ATT​GAT​TC	215bp
	R: CTG​CAA​ATG​CCT​ACA​AGC​AA	
EI	F: AGT​TGC​CCT​GGT​TTC​AAA​GA	200bp
	R: CCG​TCG​TCT​GTA​CAT​TGT​GG	
NPC2	F: TCT​TGC​AGT​TGC​CAC​TTG​AC	204bp
	R: AAT​GTT​ACC​GAT​GCC​GAG​TC	
C3	F: TTA​TCA​TGG​TCC​TGG​GTG​CT	208bp
	R: GCG​TCA​AAC​TCG​AAC​GTT​TT	
PTCH3	F: TGG​ATG​ATT​GAG​GCT​GTG​GT	180bp
	R: CCT​ACG​CAG​CCA​TTT​CCA​TC	
SMO	F: GAA​CAG​GGT​TGG​TTG​CTC​AG	174bp
	R: ATT​GAA​GGC​GCT​GCT​GTT​AG	
DHH	F: CGC​GTC​CTC​TCC​CTA​AAC​TA	161bp
	R: CCC​ACT​CCA​ACA​TTC​TCC​CT	
GLI2	F: GTG​TGT​GAA​ATG​CAG​CCT​CA	191bp
	R: GCA​TCA​CCT​GTC​AAG​TCC​AC	

**FIGURE 5:**
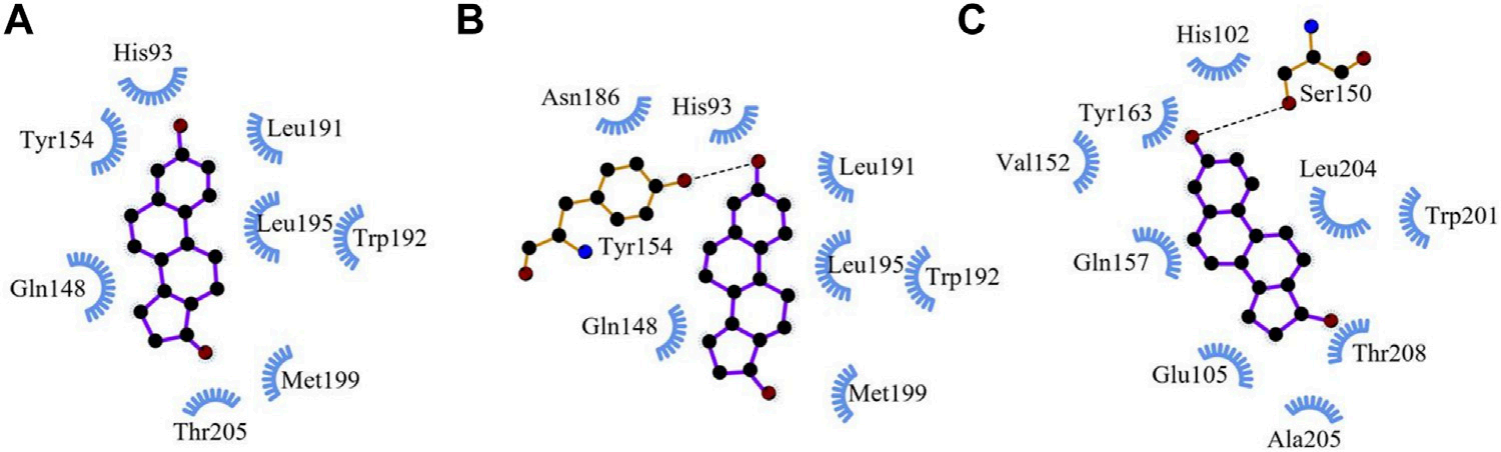
Comparison of the docking poses of 17§HSD14 with similar ligands generated using LigPlot^+^ v1.4 software (Laskowski and Swindells 2011). Circled residues indicate side chains that are involved in docking for at least two of the three models. **(A)** crystal structure of estrone bound to human 17§HSD14 (5HS6). **(B)** crystal structure of human 17bHSD14 docked to estradiol. **(C)** crystal structure of *Exaiptasia* l7§HSD14 docked to estradiol.

Since a crystal structure of 17βHSD14 with a similar ligand (estrone) is available on the PDB (5HS6), it is possible to compare the binding site of an experimentally-determined protein-ligand structure with our docked models (Laskowski and Swindells, 2011). Figure 5 shows a comparison between human 17βHSD14 with bound estrone, human 17βHSD14 with docked estradiol, and *Exaiptasia* 17βHSD14 with docked estradiol. The search space was confined to the binding pocket for each protein-ligand interaction, and the orientation of the ligand within the pocket was fairly well-conserved (see Figure 5). Additionally, the residues that interact with each ligand were well-conserved between cnidarian and homologous models. For example, estrone and estradiol formed hydrophobic contacts with His93, Gln148, Trp192, and Leu195 in both crystal structure and the docked model of human 17βHSD14. In the cnidarian protein, these residues correspond to His102, Gln157, Trp201, and Leu204, and are predicted to interact the estradiol ligand in the docked model as well (see Figure 5).

## Discussion

Attempts at detecting endocrine disruption in cnidarians has been enigmatic. Genomic studies indicate that cnidarians lack *CYP19a1a* (aka: aromatase) that vertebrates use for sex hormone steroidogenesis (Goldstone, 2008), and yet some cnidarians have demonstrated aromatase activity (Twan et al., 2003). This study sought to identify a suite of GOIs that exhibit differential transcriptional expression in anemones exposed to chemicals known to be endocrine disruptors. The EDCs used in this investigation are lipophilic and detected in the marine environment including sediments and sewage effluent in ng/L to µg/L concentrations (Atkinson et al., 2003; Singh et al., 2010; French et al., 2015; Wear and Thurber, 2015; Al-Jandal et al., 2018; Mitchelmore et al., 2019). Furthermore, all GOIs in this investigation have direct interactions or indirect associations with sterols. Using cholesterol in this investigation was critical as a positive control for evaluating GOIs binding of, and/or interactions with, cholesterol. Transcriptional activities and binding affinities of 17βHSDs in the presence of E2 and T are positive controls since these steroidal hormones are the natural ligands for 17βHSDs.

### Links to Steroidogenesis

Endocrine disruption involves chemicals that alter steroidogenesis which interfere with the hormonal signaling leading to down-stream physiological abnormalities in reproduction, development, increased risk of disease, and/or interference with immune and nervous system functions (Heindel et al., 2012). The 17β-hydroxysteroid dehydrogenases (17βHSDs) are involved with steroidogenesis of both estrogens and androgens. 17βHSDs have been detected in cnidarians (Tarrant et al., 2003; Blomquist et al., 2006; Shikina et al., 2016). *17βHSD12* and *17βHSD14* represent the last step in the steroidogenesis pathway prior to the aromatization of T into E2. *17βHSD12* reduces estrone (E1) into E2 as well as androstenedione (A4) into T, while *17βHSD14* performs the opposite reaction by oxidizing E2 into E1, and T into A4 (Luu-The et al., 2006; Caldwell et al., 2012; Sivik et al., 2012; Thomas and Potter, 2013; Shikina et al., 2016; Salah et al., 2019). Concentrations of competing sterols may drive which sterol gets converted by a specific 17βHSD (Lathe and Kotelevtsev, 2014). E2 concentration is known to regulate transcription of 17βHSDs (Rotinen et al., 2009). Transcriptional profiles herein demonstrate the organism (regardless of sex) responds to the environmental “overload” of E2 or T by modulating the transcription of the enzyme in order to transform both sex hormones into less potent sterols. These profiles also reveal that cholesterol and xenobiotics (BBP and BP-3) interfere with steroidogenesis by altering normal transcriptional demands for 17βHSDs (see Figure 1).

### Links to Sterol Binding and Transport

Cnidarians get cholesterol and other sterols by either dietary intake and/or from their endosymbiont (Revel et al., 2016; Hambleton et al., 2019). Cholesterol transport and metabolism is tightly regulated (Maxfield et al., 2016). Due to lipid-rich tissues, it has been proposed that lipophilic compounds will easily diffuse into cnidarian tissues (Peters et al., 1997). Sterols are most concentrated in cnidarian epidermis and some sterols are known to increase in concentration after exposure to environmental pollutants (Revel et al., 2016; Stien et al., 2020). The lack of significant expression of *C3* in the cholesterol treatment is consistent with the fact that it is a natural constituent of animal membranes.

Five GOIs (*C3, PTCH3, CTSL, EI, NPC2*) have functional associations with the endocytic processes whereas a sixth GOI (*DHH*) is a secreted morphogen associated with exocytosis/secretion. *C3* is associated with phagocytosis of foreign particles into cnidarians (Palmer and Traylor-Knowles, 2012), while PTCH regulates functions of: receptor-mediated endocytosis of sterol-protein ligands; down-stream morphogens; and the removal of oxysterols (Incardona et al., 2000; Strutt et al., 2001; Corcoran and Scott, 2006; Zhong et al., 2014). Three GOIs (*CTSL, EI, NPC2*) are associated with lysosomes and phagocytosis. Cathepsins are lysosomal proteases and *EI* is the cnidarian homolog of an inhibitor of cathepsins (Lenarčič et al., 1997; Štrukelj et al., 2000). Lysosomes contribute to the termination of hormone effects by degrading proteins interacting with the hormone (Qualmann et al., 2000; Totta et al., 2014). NPC2 functions as a key regulator of sterol transport between *Symbiodinium* and cnidarian hosts which is representative of stable symbioses in a structure known as the symbiosome (Infante et al., 2008; Vance, 2010; Ganot et al., 2011; Dani et al., 2014; Bucher et al., 2016; Revel et al., 2016; Hambleton et al., 2019; Lu et al., 2020). Seven GOIs (*NPC2, 17βHSD14, C3, PTCH3, SMO, DHH, VTG*) have known linkages to endoderm. (Kvennefors et al., 2010; Shikina et al., 2013; Sanders et al., 2014; Shikina et al., 2016).

Five GOIs (*17βHSD14, 17βHSD12, NPC2, PTCH3, SMO*) that have sterol binding pockets were used in *silico* modelling. *In silico* modelling has previously demonstrated phthalates and steroids without an aromatic A-ring are capable of binding to sterol-binding proteins (Sheikh and Beg, 2017; Khalturin et al., 2018). Patched genes have multiple functionally redundant homologs that cannot discriminate between ligands (Carpenter et al., 1998; Zhulyn et al., 2015; Hu and Song, 2019). Justification for model docking is based on the fact that cnidarian genomes have representative vertebrate homologs of relevant proteins (Miller et al., 2007; Putnam et al., 2007; Kvennefors et al., 2010). *In silico* modelling demonstrates the lowest energy docking poses for the crystal structures of the homologous proteins and the models of the *Exaiptasia* proteins correlate well with each other (see Table 3). The binding scores for the *Exaiptasia* protein models generally follow the same trends as the homologs from the PDB. In both cases, the steroid ligands (cholesterol, E2, and T) bound more strongly than BP-3 or BBP, although the differences in binding energy seem to be smaller for the 17βHSD12 and 17βHSD14 proteins. This result was expected, since BBP and BP-3 are considerably smaller and as such, would make less contact with binding pocket residues (see Table 3). Although there are a few minor differences between homologs, such as hydrogen bonding patterns, the orientation of the ligand in the 17βHSD14 binding pocket and the side chains involved in binding are strikingly similar (see Figure 5). All sterols have similarities in orientations, binding affinities, and expression profiles (see Table 3 and Figure 5). 17βHSDs are known to exhibit variability in substrate binding (Blomquist et al., 2006; Lathe and Kotelevtsev, 2014).

Data herein provides new information about the diversity of ligands, including xenobiotics that bind to NPC2, PTCH3, SMO, and the 17βHSDs. The differential expression of these sterol-binding proteins suggests that these proteins handle natural sterols and xenobiotics differently. Laboratory-induced exposure to cholesterol, E2, and T requires modulating transcript copy numbers to handle the environmental flux of natural ligands. However, laboratory-induced exposures of BBP and BP-3 appear to require transcriptional replacement for proteins after binding xenobiotics. All GOIs except *SMO* exhibit up-regulated expression in the BP-3 and BBP treatments (see Figures 4E,F).

### Links to Development

Hedgehog (HH) signaling is a highly conserved developmental pathway that relies on cholesterol interacting with specific sterol-binding proteins. Endocrine disruption is known to interfere with HH signaling (Johansson and Svingen, 2020). Developmentally-regulated signaling pathways involving apoptosis, embryogenesis, cell proliferation, and development of diseases present in higher animals are also found in cnidarians including *Exaiptasia* (Technau et al., 2005; Matus et al., 2008; Sinkovics, 2015; Bucher et al., 2016). Four GOIs (*PTCH3, SMO, DHH, GLI2*) can be linked to HH signaling. E2 acts as a direct HH pathway antagonist (Lipinski and Bushman, 2010) and the E2 expression profile in Figure 3 reaffirms this inhibitory effect. Recognized inhibitors of HH signaling also include the well-characterized steroidal alkaloid cyclopamine (Chen 2002). The down-regulated expression profiles of *PTCH3* in the E2, T, and cholesterol treatments and the down-regulated expression of *SMO* in the T and cholesterol treatments suggests all three sterols exhibit similar inhibitory effects (see Figure 3). The HH pathway is also responsive to xenobiotics as well as sterols (Johansson and Svingen, 2020). Phthalates are known to impact DHH and PTCH expression, and BP-3 is considered estrogenic (Thibaut and Porte, 2004; Borch et al., 2006; Downs et al., 2016; Hong et al., 2016; LaPlante et al., 2018; Matouskova et al., 2020). Although PCTH3 and DHH were down-regulated in response to sterols, *PTCH3*, *DHH*, and *GLI2* were up-regulated in response to BP-3 and BBP (see Figure 3), suggesting opposing effects of sterols and xenobiotics on HH signaling, presumably with sterols exerting antagonists effects and xenobiotics exerting agonist effects. Regardless, the expression profiles in Figure 3 highlight the inherent vulnerability of the HH pathway to diverse chemical signals.

### Links to Gametogenesis

Gametogenesis is associated with HH signaling. HH signaling molecules are considered stem-cell factors (Zhang and Kalderon, 2001). Evidence exists in other metazoans that secreted molecules in the HH signaling pathway are one of the primary regulators of stem cells which are necessary precursors of oogenesis (Zhang and Kalderon, 2001). Shikina and Chang (2016) identifies cnidarian germline stem cells as originating in endodermal mesentery. HH signaling GOIs (*PTCH3, SMO, DHH*) are known to be restricted to the endoderm of male and female gonophores (Sanders et al., 2014). Germline stem cells are the essential cells responsible for this process in various metazoans. In *Drosophila*, somatic cells in the ovary of cannot proliferate normally in the absence of Smoothened activity (Zhang and Kalderon, 2000), and excessive HH signaling can result from aberrant expansion of stem cell pools (Zhang and Kalderon, 2001). Results show all exogenous treatments (except E2) caused down-regulation of *SMO* (see Figure 4). With the exception of *PTCH3*, results also show that HH signaling GOIs in the E2 treatment are not significantly expressed (see Figure 3).

*Exaiptasia* reproductive strategies include both sexual and asexual reproduction (Clayton, 1985; Grawunder et al., 2015). Subsets of anemones used for each treatment were taken from the same population as the control anemones. Although not confirmed, it is highly likely that all anemones used in this investigation were clonal. Detecting the presence of sex-specific tissues enhances the ability to interpret anemone responses to the EDCs. Although the sex of the colony was not known, expression profiles of the GOIs could provide clues about the sex of these animals. Vitellogenin is a female-specific protein commonly found across the animal kingdom and is the most abundant protein associated with oocyte development in cnidarians (Shikina et al., 2013; Levitan et al., 2015). Hormonal and/or environmental cues are known to stimulate release of oocytes (Levitan et al., 2015). Exogenous E2 is known to influence pedal laceration and egg bundle formation in cnidarians (Tarrant et al., 2004; Thorn et al., 2015). Blue-light cues have also been used to induce *Aiptasia sp* gametogenesis in a laboratory setting (Grawunder et al., 2015).

This investigation also did not attempt to identify the sex of the anemones nor their reproductive status, per se. Hermaphroditism and even Trioecy has been observed in closely related species (Schlesinger et al., 2010; Armoza-Zvuloni et al., 2014). A small amount of data from a previous study suggests male tissues from hermaphroditic corals also express *VTG* (Hayakawa et al., 2005). Results reveal *VTG* expression in the control treatment suggesting anemones are female and already transcribing *VTG* (see Figure 1). Decreases in vitellogenin in females can be useful for identifying EDCs (Ankley et al., 2002). If these anemones were male, then up-regulation of vitellogenin would have been expected. Up-regulation of vitellogenin in males is widely recognized as the prominent estrogenic effect in many aquatic animals exposed to exogenous E2 (Denslow et al., 1999; Matozzo et al., 2008). These results cannot confirm nor reject the possibility that male reproductive tissues were also present.

A second piece of evidence suggest that these anemones were female was the expression of *17βHSD14* in E2 which was significantly up-regulated (*p* < 0.0001) compared to the T, cholesterol, BBP, and BP-3 treatments. Female anemones perform oogenesis which is stimulated by the presence of E2 so when exogenous E2 is introduced, additional *17βHSD14* is required to reduce E2 potency by transformation into E1. In this investigation, *VTG* expression was crucial for differentiating E2 exposure from other EDC exposures. The expression profiles of *17βHSD14* and *17βHSD12* can be indirect measures of cellular demands for sex hormones. When exposed to exogenous sex hormones, transcription of *17βHSD14* is up-regulated while *17βHSD12* is not significantly different from the control. These results suggest these anemones were females responding to the EDCs in this investigation. Future investigations using these GOIs, coupled with lab-controlled methodologies to induce gametogenesis in recognized clonal lines of anemones should provide greater resolution in how these organisms respond to EDCs.

## Conclusion

This data demonstrates that endocrine disrupting exogenous sterols (E2, T, cholesterol) and xenobiotics (BP-3, BBP) alter transcription of genes associated with steroidogenesis, sterol transport, oogenesis, and the Hedgehog signaling pathway (Figures 1–4). *In silico* modelling demonstrates that EDC’s bind favorably into pockets of proteins involved with steroidogenesis, cholesterol transport, and HH signaling. Because these GOIs were expressed at basal levels in the control treatment (i.e. unstressed conditions) and significantly differentially expressed when exposed to EDCs (Figure 4), they may serve as transcriptional biomarkers. Biomarkers have the potential to link field and laboratory studies by acting as ‘‘mechanistic signposts’’ of exposure to anthropogenic chemicals (Hutchinson, 2007; Traylor-Knowles and Palumbi, 2014). The most validated biomarker of estrogenic exposure is *VTG*, an estrogen-dependent glycolipophosphoprotein naturally expressed only in oviparous females during the reproductive season (Verderame and Scudiero, 2017). The biomarkers used in this investigation offer new insight into BBP and BP-3 exposures. Understanding mechanisms of action for xenobiotics can be advanced by utilizing known biochemical pathways involving cholesterol and sex hormones.

Collectively, the expression profiles of this suite of GOIs are capable of discriminating seven distinct outcomes. 1) It is possible to discern differences between control vs exposure to exogenous chemicals (i.e. unstressed vs stressed) (see Figures 1–4). 2) Expression profiles of GOIs involved with HH signaling can differentiate E2 exposure from other sterols (see Figure 3). 3) Cholesterol exposure can be discriminated from sex hormones by expression of *C3* (see Figures 4B–D). 4) *C3* expression can also differentiate BP-3 versus BBP exposure (see Figure 2). 5) It is possible to differentiate natural sterols from xenobiotic exposure (see Figure 4). 6) Expression profiles of *VTG* and *17βHSD 12/14* provide a mechanism for discerning the responses of female reproductive tissues in E2 exposure compared to T or cholesterol exposures (see Figure 1). 7) Expression of *17βHSD14* and *17βHSD12* were critical for discerning BBP and BP-3 treatments compared to exogenous sterols (see Figure 1). Coupled with expression profiling, *in silico* results reveal proteins that bind cholesterol or modify sex hormones are vulnerable to endocrine disruption in the presence of either xenobiotics or exogenous sterols.

Future ecotoxicological investigations using more sensitive analytical measurements of chemical concentrations should enable identifying minimal concentrations necessary to alter expression of these GOIs. A primary goal of this investigation was to identify GOIs that are responsive to these EDCs. Moving forward, future work can focus on measuring GOI responses over recognized acute toxicity timeframes such as 24–96 hrs. Lastly, it will also be important to establish the baseline temporal variance in GOI expression before, during, and after the peak reproductive season.

In summary, this investigation provides new insight into anemone responsiveness to a small set of chemicals recognized as endocrine disruptors. Individually, each GOI provides a clue about a specific biochemical pathway, cellular process, and/or developmental pathway. Collectively, these GOIs offer greater resolution into understanding how anemones respond to different EDCs. More than a decade ago, Tarrant (2007) questioned if sex hormones are disrupting or even impacting cnidarians and the enigma of aromatase remains. However, the data herein offers new information towards deciphering endocrine disruption in cnidarians.

## Data Availability

The original contributions presented in the study are included in the article/Supplementary Material, further inquiries can be directed to the corresponding author.
